# *Mycobacterium avium* complex genomics and transmission in a London hospital

**DOI:** 10.1183/13993003.01237-2022

**Published:** 2023-04-20

**Authors:** Andries J. van Tonder, Huw C. Ellis, Colin P. Churchward, Kartik Kumar, Newara Ramadan, Susan Benson, Julian Parkhill, Miriam F. Moffatt, Michael R. Loebinger, William O.C. Cookson

**Affiliations:** 1 Department of Veterinary Medicine, University of Cambridge, Cambridge, UK; 2 Host Defence Unit, Department of Respiratory Medicine, Royal Brompton Hospital, Guy's and St Thomas’ NHS Foundation Trust, London, UK; 3 National Heart and Lung Institute, Imperial College London, London, UK; 4 Department of Microbiology, Royal Brompton Hospital, Guy's and St Thomas’ NHS Foundation Trust, London, UK; 5 These three authors contributed equally

## Abstract

**Background:**

Non-tuberculous mycobacteria (NTM) are environmental microorganisms and opportunistic pathogens in individuals with pre-existing lung conditions such as cystic fibrosis (CF) and non-CF bronchiectasis. While recent studies of *Mycobacterium abscessus* have identified transmission within single CF centres as well as nationally and globally, transmission of other NTM species is less well studied.

**Methods:**

To investigate the potential for transmission of the *Mycobacterium avium* complex (MAC) we sequenced 996 isolates from 354 CF and non-CF patients at the Royal Brompton Hospital (London, UK; collected 2013–2016) and analysed them in a global context. Epidemiological links were identified from patient records. Previously published genomes were used to characterise global population structures.

**Results:**

We identified putative transmission clusters in three MAC species, although few epidemiological links could be identified. For *M. avium*, lineages were largely limited to single countries, while for *Mycobacterium chimaera*, global transmission clusters previously associated with heater-cooler units (HCUs) were found. However, the immediate ancestor of the lineage causing the major HCU-associated outbreak was a lineage already circulating in patients.

**Conclusions:**

CF and non-CF patients shared transmission chains, although the lack of epidemiological links suggested that most transmission is indirect and may involve environmental intermediates or asymptomatic carriage in the wider population.

## Abstract 2

**Transmission of *Mycobacterium avium* complex (MAC) species is indirect and may involve environmental intermediates or asymptomatic carriage in the wider population** https://bit.ly/3FQWqnF

## Introduction

Non-tuberculous mycobacteria (NTM) are ubiquitous environmental microorganisms found in soil and water, and are considered opportunistic pathogens in humans. Individuals with pre-existing genetic or acquired lung diseases such as cystic fibrosis (CF), non-CF bronchiectasis and COPD are more prone to NTM disease, although individuals with no known immune dysfunction can also present with NTM infections [1–3]. Globally, disease due to NTM infections is increasing in prevalence; the estimated prevalence of NTM disease in the USA rose from 2.4 per 100 000 in the early 1980s to 15.2 per 100 000 in 2013 [4]. NTM infections may be progressive and treatment requires prolonged multidrug therapy [5]. Treatment is often unsuccessful due to an absence of antimicrobial agents with low toxicity and effective *in vivo* activity against NTM species [1].

A number of NTM species including *Mycobacterium abscessus* and members of the *Mycobacterium avium* complex (MAC), notably *M. avium* and *Mycobacterium intracellulare*, have emerged as major respiratory pathogens in the past three decades [6–8]. Another member of the MAC, *Mycobacterium chimaera*, has also been implicated in numerous global infections associated with cardiothoracic surgery, with the source of infections linked to LivaNova heater-cooler units (HCUs) contaminated during their manufacture [9–11].

Until recently the prevailing hypothesis was that NTM infections were due to independent acquisitions from environmental sources such as soil, contaminated drinking water distribution systems and household plumbing [12, 13]. Recent studies of *M. abscessus* in CF patients have, however, identified indirect patient–patient transmission within a single CF centre as well as the presence of globally circulating clones of *M. abscessus* among CF patients worldwide [14–18]. An observational study of *M. abscessus* across England showed that these dominant clones are also found in patients with other chronic respiratory diseases, but was unable to identify epidemiological links for most closely related isolates, suggesting environmental acquisition [19]. However, a wider analysis demonstrated that transmission networks involve both people with CF and those without, and that these networks are global. It is therefore likely that transmission is complex, involving multiple patient cadres as well as environmental intermediates [20]. In the case of *M. chimaera*, a global genetic analysis of 250 isolates from patients, HCUs and the factory of origin suggested a point-source contamination during manufacture causing global distribution followed by localised transmission [11].

While transmission of MAC between CF patients in the USA has been investigated [21], little work has been done to examine whether transmission of MAC occurs between patients with CF, non-CF bronchiectasis or other chronic respiratory diseases. Using a large collection of longitudinal isolates collected from patients attending the Royal Brompton Hospital (London, UK), the aims of this study were to characterise the population structure of MAC, to identify potential transmission chains involving patients with CF and other non-CF lung conditions, and to place the Royal Brompton Hospital isolates in a global context.

## Methods

### Bacterial isolates and study period

Isolates were collected from 363 patients attending the respiratory inpatient and outpatient clinics of the Royal Brompton Hospital between January 2013 and April 2016. DNA extractions were performed on confirmed MAC cultures and the Illumina Hiseq X10 platform (Illumina, San Diego, CA, USA) was used to generate 2×150 bp paired-end reads (supplementary text S1). Sequencing reads were deposited in the European Nucleotide Archive under project PRJEB21813 (supplementary file S2).

### Whole-genome sequencing and data analysis

Following sample quality control, whole-species maximum likelihood phylogenetic trees were built with IQ-TREE version 1.6.5 using a previously described pipeline [22] (supplementary text S1), including published sequences for context (supplementary file S3). Pairwise single nucleotide polymorphism (SNP) distances for within-patient longitudinal isolates for each species in the Royal Brompton Hospital datasets were used to calculate thresholds for defining transmission clusters (supplementary text S1).

## Results

### Patient demographics

In total, 354 patients were included in the study. The clinical and demographic characteristics of the study population are shown in table 1.

**TABLE 1:** Patient clinical and demographic data: all patients (n=354)

**Male**	174 (49.2)
**Age at first culture (years)**	56 (5–93)
**BMI at first culture (kg·m^−2^)**	22.5 (13.4–43.4)
**Current or ex-smoker**	28 (10.7)
**Underlying pulmonary disease**	
None	12 (3.4)
Non-CF bronchiectasis	147 (41.5)
CF	87 (24.6)
COPD	53 (15.0)
Asthma	32 (9.0)
Allergic bronchopulmonary aspergillosis	19 (5.4)
Interstitial lung disease	17 (4.8)
Other (pleural thickening, sarcoidosis)	7 (2.0)
**Antibiotic treatment during study period**	55 (15.5)
**Clinical data unavailable**	20 (5.6)

Data are presented as n (%) or median (range). BMI: body mass index; CF: cystic fibrosis.

### Species distribution

**FIGURE 1:**
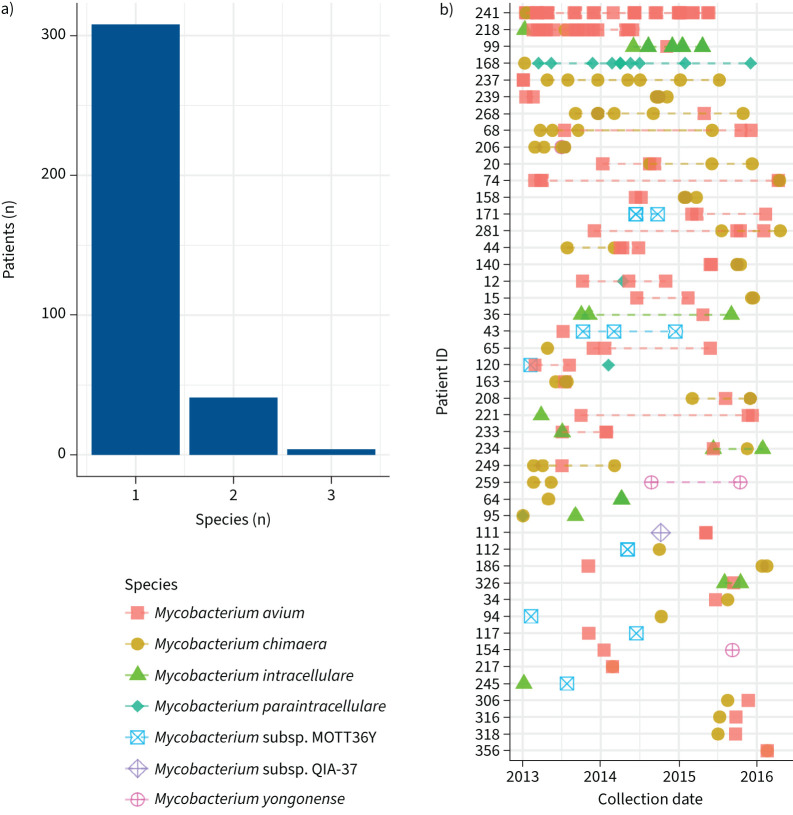
Patients infected by more than one *Mycobacterium avium* complex (MAC) species. a) Histogram showing the number of MAC species identified in each patient. b) Timeline of patients infected by more than one MAC species. Each point represents an isolate and is coloured according to the species identified. Isolates from the species in a patient are linked together. Patients with one isolate or multiple isolates from a single species were excluded.

996 isolates from 354 patients from nine MAC species were sequenced (table 2). Three species accounted for 926 of the 996 of MAC isolates (93.0%): *M. avium* (*M. avium* subsp. *avium* and *M. avium* subsp. *hominissuis*), *M. chimaera* and *M. intracellulare.* Most patients were infected with only a single species during the collection period. However, 45 of the 354 patients (12.7%) were infected with two or more species (figure 1a). In this group, most of the isolates collected were typically from a single species, with other species observed more infrequently (figure 1b). Subsequent analyses in this study will focus on the three predominant species in the dataset: *M. intracellulare*, *M. avium* (*M. avium* subsp. *avium* and *M. avium* subsp. *hominissuis*) and *M. chimaera*.

**TABLE 2:** *Mycobacterium avium* complex species identified in the Royal Brompton Hospital collection

**Species**	**Genomes (n)**
***Mycobacterium avium***	405
***Mycobacterium chimaera***	359
***Mycobacterium intracellulare***	162
***Mycobacterium* sp. MOTT36Y**	39
***Mycobacterium paraintracellulare***	15
***Mycobacterium yongonense***	10
***Mycobacterium marseillense***	4
***Mycobacterium colombiense***	1
***Mycobacterium* sp. QIA-37**	1
**Total**	996

### M. intracellulare

**FIGURE 2:**
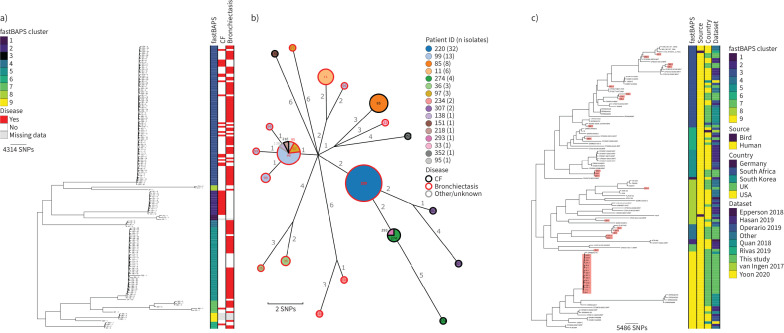
Population structure and transmission of *Mycobacterium intracellulare*. a) Midpoint-rooted maximum likelihood phylogenetic tree for *M. intracellulare* isolates collected at the Royal Brompton Hospital (n=162). The taxa are clustered according to their sequence similarities. The lengths of the branches are scaled in nucleotide substitutions per site. The disease status of the patients (that isolates were collected from) for cystic fibrosis (CF) and non-CF bronchiectasis is represented by red bars for “Yes” and white bars for “No”, with missing patient data shown by grey bars. Scale bar shown in single nucleotide polymorphisms (SNPs) per site. b) *M. intracellulare* Mi_FB3_1 transmission cluster. Each node represents an isolate or isolates identical (0 SNPs) to each other and the size of the node is proportional to the number of identical isolates. Nodes are coloured and labelled by patient ID. The outer ring of each node is coloured according to the disease status of the patient. The edges represent the pairwise SNP distance between the isolate(s). c) Midpoint-rooted maximum likelihood phylogenetic tree for global *M. intracellulare* isolates (n=114). The taxa are clustered according to their sequence similarities. The lengths of the branches are scaled in nucleotide substitutions per site. fastBAPS lineage, source of isolate, country of collection and study are shown as datastrips to the right of the phylogenies. Isolates from the Royal Brompton Hospital are highlighted in salmon colour. Scale bar shown in SNPs per site. Details of the study datasets are provided in supplementary text S1 and supplementary file S3.

162 genomes from 37 patients were identified as *M. intracellulare* (figure 2a). 11 patients had CF, 17 had non-CF bronchiectasis and seven had other lung conditions (COPD n=3; interstitial lung disease (ILD) n=3; asthma n=1; congenital pulmonary airway malformation n=1). Disease metadata were missing for two patients. Genomic clustering with fastBAPs identified nine lineages, with three of these having more than 10 genomes (figure 2a). Following remapping to local references for the three largest lineages, three putative transmission clusters were identified, with the largest, Mi_FB3_1, composed of 16 patients (figure 2b and supplementary table S2). Of these 16 patients, eight had non-CF bronchiectasis, seven had CF and one had ILD. During the sampling period, four patients were treated with antibiotics, with successful outcomes in three patients. No epidemiological links were identified between patients in the year prior to isolate collection. Comparison with 77 previously published *M. intracellulare* isolates showed that the most closely related contextual isolate was collected in the UK in 2015/2016 [23] and formed part of a clade containing isolates from the Mi_FB3_1 cluster (figure 2c).

### M. avium

405 sequenced isolates collected from 176 patients were identified as *M. avium*. Sequence data for all *M. avium* isolates were mapped to a single reference (*M. avium* subsp. *avium* 104; NC008595.1) and a phylogenetic tree constructed with *M. avium* subsp. *paratuberculosis* (DRR263663) as an outgroup (supplementary figure S2). The structure of this phylogeny showed that there were two major clades which corresponded to the two subspecies *M. avium* subsp. *avium* (n=207) and *M. avium* subsp. *hominissuis* (n=198). Each of these subspecies was analysed separately.

#### *M. avium* subsp. *avium*

**FIGURE 3:**
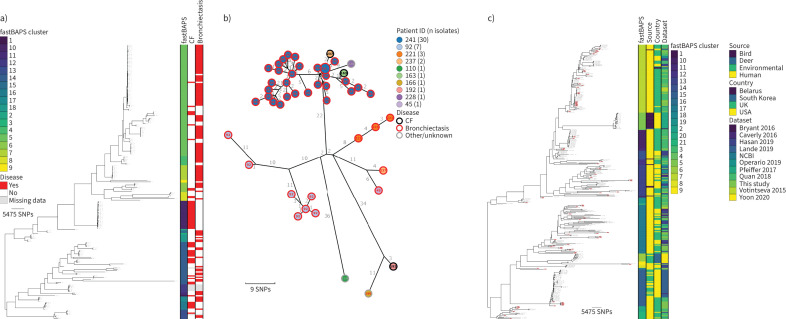
Population structure and transmission of *Mycobacterium avium* subsp. *avium*. a) Midpoint-rooted maximum likelihood phylogenetic tree for *M. avium* subsp. *avium* isolates collected at the Royal Brompton Hospital (n=207). The taxa are clustered according to their sequence similarities. The lengths of the branches are scaled in nucleotide substitutions per site. The disease status of the patients (that isolates were collected from) for cystic fibrosis (CF) and non-CF bronchiectasis is represented by red bars for “Yes” and white bars for No, with missing patient data shown by grey bars. Scale bar shown in single nucleotide polymorphisms (SNPs) per site. b) *Mycobacterium avium* subsp. *avium* MAA_FB5_1 transmission cluster. Each node represents an isolate or isolates identical (0 SNPs) to each other and the size of the node is proportional to the number of identical isolates. Nodes are coloured and labelled by patient ID. The outer ring of each node is coloured according to the disease status of the patient. The edges represent the pairwise SNP distance between the isolate(s). c) Midpoint-rooted maximum likelihood phylogenetic tree for global *M. avium* subsp. *avium* isolates (n=344). The taxa are clustered according to their sequence similarities. The lengths of the branches are scaled in nucleotide substitutions per site. fastBAPS lineage, source of isolate, country of collection and study are shown as datastrips to the right of the phylogenies. Isolates from the Royal Brompton Hospital are highlighted in salmon colour. Scale bar shown in SNPs per site. Details of the study datasets are provided in supplementary text S1 and supplementary file S3. NCBI: National Center for Biotechnology Information.

Of the 76 patients infected with *M. avium* subsp. *avium*, 15 had CF, 39 had non-CF bronchiectasis and 16 had other lung conditions (COPD n=10; ILD n=3; allergic bronchopulmonary aspergillosis (ABPA) n=3; asthma n=6), while three patients, including one smoker, had no pre-existing respiratory disease (metadata were unavailable for three patients) (figure 3a). Clustering of the 207 *M. avium* subsp. *avium* genomes identified 18 fastBAPS lineages (figure 3a). Remapping of the four largest lineages allowed the identification of seven putative transmission clusters comprising between two and 10 patients (supplementary table S3), with the largest cluster of 10 isolates comprised of patients with CF (n=2), non-CF bronchiectasis (n=6) and asthma or COPD (n=2) (figure 3b).

**FIGURE 4:**
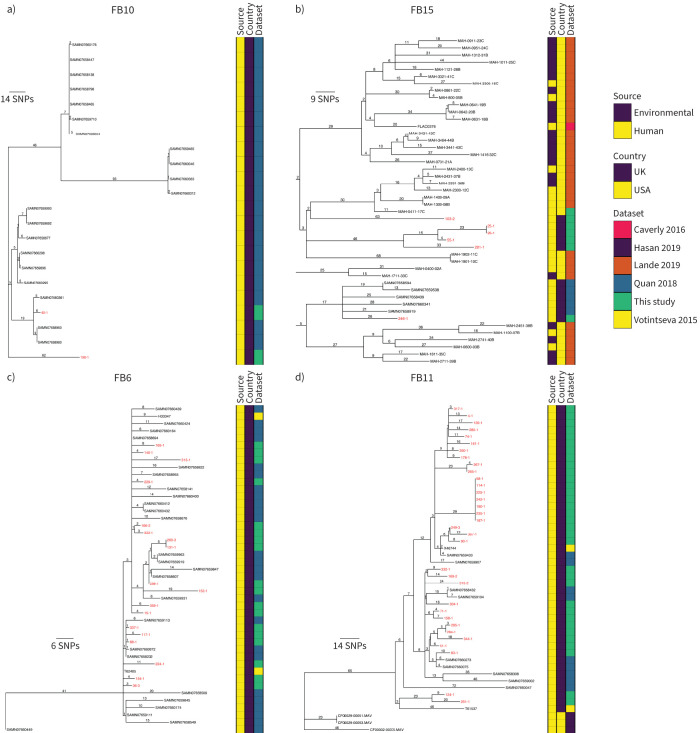
*Mycobacterium avium* global fastBAPS phylogenies. Midpoint-rooted maximum likelihood phylogenetic trees for a) *M. avium* subsp. *avium* FB10, b) *M. avium* subsp. *avium* FB15, c) *M. avium* subsp. *hominissuis* FB6 and d) *M. avium* subsp. *hominissuis* FB11. The taxa are clustered according to their sequence similarities. The lengths of the branches are scaled in nucleotide substitutions per site. The source of isolate, country of collection and study are shown as datastrips to the right of the phylogenies. Isolates from the Royal Brompton Hospital are shown in red colour. Scale bars shown in single nucleotide polymorphisms (SNPs) per site. Details of the study datasets are provided in supplementary text S1 and supplementary file S3.

21 fastBAPS lineages were defined in the global phylogeny (figure 3c). Examination of the distribution of pairwise SNP distances for each lineage containing at least 10 genomes revealed that there were two lineages, MAA_FB10 and MAA_FB15, containing Royal Brompton Hospital isolates with lower median pairwise SNP distances (figure 3c and supplementary figure S3b). The 23 genomes in FB10 were collected in the UK between 2013 and 2016, with most coming from the National Mycobacterial Reference Service which characterises mycobacterial cultures from across the Midlands and North of England (figure 4a) [23]. If the SNP threshold used to calculate putative transmission clusters circulating in the Royal Brompton Hospital was applied, then one isolate from the Royal Brompton Hospital (40-1) formed part of a cluster comprised of isolates from Quan *et al.* [23]. The majority (35 out of 46) of the isolates forming MAA_FB15 were collected in the USA, with most from a study investigating *M. avium* in the community and household water in Philadelphia (figure 4b) [24]. The pairwise distances between two of the Royal Brompton Hospital isolates and other UK isolates and isolates from the USA in MAA_FB15 were under our threshold, suggesting potential cross-Atlantic transmission (supplementary figure S4a).

#### *M. avium* subsp. *hominissuis*

**FIGURE 5:**
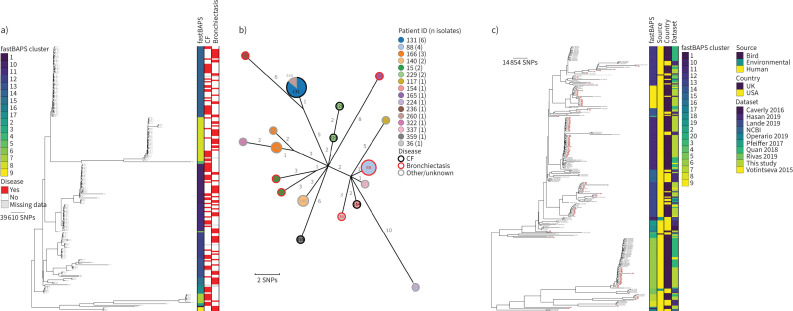
Population structure and transmission of *Mycobacterium avium* subsp. *hominissuis*. a) Midpoint-rooted maximum likelihood phylogenetic tree for *M. avium* subsp. *hominissuis* isolates collected at the Royal Brompton Hospital (n=198). The taxa are clustered according to their sequence similarities. The lengths of the branches are scaled in nucleotide substitutions per site. The disease status of the patients (that isolates were collected from) for cystic fibrosis (CF) and non-CF bronchiectasis is represented by red bars for “Yes” and white bars for “No”, with missing patient data shown by grey bars. Scale bar shown in single nucleotide polymorphisms (SNPs) per site. b) *M. avium* subsp. *hominissuis* MAH_FB8_1 transmission cluster. Each node represents an isolate or isolates identical (0 SNPs) to each other and the size of the node is proportional to the number of identical isolates. Nodes are coloured and labelled by patient ID. The outer ring of each node is coloured according to the disease status of the patient. The edges represent the pairwise SNP distance between the isolate(s). c) Midpoint-rooted maximum likelihood phylogenetic tree for global *M. avium* subsp. *hominissuis* isolates (n=236). The taxa are clustered according to their sequence similarities. The lengths of the branches are scaled in nucleotide substitutions per site. fastBAPS lineage, source of isolate, country of collection and study are shown as datastrips to the right of the phylogenies. Isolates from the Royal Brompton Hospital are highlighted in salmon colour. Scale bar shown in SNPs per site. Details of the study datasets are provided in supplementary text S1 and supplementary file S3. NCBI: National Center for Biotechnology Information.

198 genomes from 106 patients were characterised as *M. avium* subsp. *hominissuis* (figure 5a). 33 patients had CF, 43 had non-CF bronchiectasis and 21 had other lung conditions (COPD n=14; ILD n=1; ABPA n=5; sarcoidosis n=1; asthma n=11). Four patients had no pre-existing respiratory disease. Disease metadata were unavailable for the remaining five patients. Genomic clustering identified 17 lineages and seven putative transmission clusters containing between two and 16 patients (supplementary table S4). The largest cluster of 16 isolates is shown in figure 5b, and comprised patients with CF (n=4), non-CF bronchiectasis (n=7), COPD (n=3) and a single patient with no pre-existing lung condition (metadata were unavailable for one patient). Two pairs of patients in each of the clusters, MAH_FB8_1 and MAH_FB14_9, were found to have epidemiological links.

Of the 20 defined fastBAPS lineages in the global collection, MAH_FB6 and MAH_FB11 had lower median pairwise SNP distances and contained Royal Brompton Hospital isolates (figure 5c and supplementary figure S3c). Both lineages were composed completely or near-completely of isolates collected in the UK (figure 4c and d). The star-like structure of the FB6 phylogeny is suggestive of a point-source outbreak, and applying a transmission SNP threshold of 16 SNPs showed that 41 out of 45 of the isolates in FB6 would have formed a transmission cluster that included both isolates from this study and isolates from other UK studies (supplementary figure S4b) [23, 25]. Two small transmission clusters, containing Royal Brompton Hospital and other UK isolates, were also identified in FB11.

### M. chimaera

**FIGURE 6:**
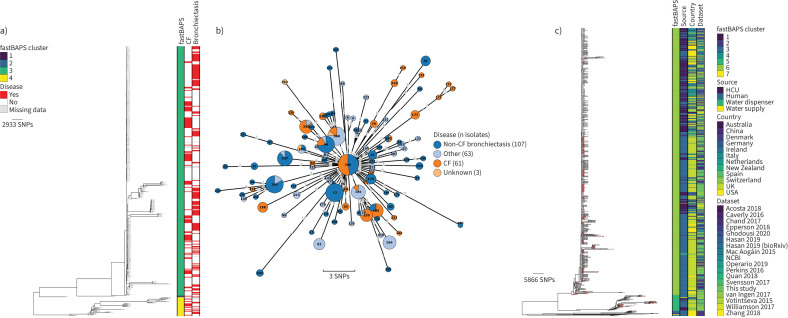
Population structure and transmission of *Mycobacterium chimaera.* a) Midpoint-rooted maximum likelihood phylogenetic tree for *M. chimaera* isolates collected at the Royal Brompton Hospital (n=359). The taxa are clustered according to their sequence similarities. The lengths of the branches are scaled in nucleotide substitutions per site. The disease status of the patients (that isolates were collected from) for cystic fibrosis (CF) and non-CF bronchiectasis is represented by red bars for “Yes” and white bars for “No”, with missing patient data shown by grey bars. Scale bar shown in single nucleotide polymorphisms (SNPs) per site. b) *M. chimaera* Mc_FB3_1 transmission cluster. Each node represents an isolate or isolates identical (0 SNPs) to each other and the size of the node is proportional to the number of identical isolates. Nodes are coloured according to patient disease status and labelled by patient ID. The edges represent the pairwise SNP distance between the isolate(s). c) Rooted maximum likelihood phylogenetic tree for global *M. chimaera* isolates (n=826). The taxa are clustered according to their sequence similarities. The lengths of the branches are scaled in nucleotide substitutions per site. fastBAPS lineage, source of isolate, country of collection and study are shown as datastrips to the right of the phylogenies. Isolates from the Royal Brompton Hospital are highlighted in salmon colour. Scale bar shown in SNPs per site. Details of the study datasets are provided in supplementary text S1 and supplementary file S3. HCU: heater-cooler unit; NCBI: National Center for Biotechnology Information.

359 sequenced isolates collected from 155 patients were identified as *M. chimaera*; 37 of the patients had CF, 60 had non-CF bronchiectasis and 50 patients had other lung conditions (COPD n=24; ILD n=10; ABPA n=11; pleural thickening with enfolded lung n=1; lung non-mucinous adenocarcinoma n=1; granulomatosis with polyangiitis n=1; *M. tuberculosis* in lymph nodes during same endoscopy procedure n=1; primary ciliary dyskinesia n=1; asthma n=17). There were five patients with no pre-existing respiratory disease and metadata were unavailable for a further nine patients. A single lineage, Mc_FB3, corresponding to the previously characterised Group 1 accounted for 332 out of 359 (92.5%) of the sequenced isolates, with most of the remaining isolates (25 out of 359 (7.0%)) forming a second main lineage, Mc_FB4, corresponding to Group 2 (figure 6a). 13 putative transmission clusters containing between two and 106 patients were identified (supplementary table S5). 12 potential epidemiological links between 15 different patients were found in transmission cluster Mc_FB3_1, the largest transmission cluster of 106 patients (figure 6b, and supplementary tables S5 and S6). Of the patients in this cluster, 43 had non-CF bronchiectasis, 24 had CF, 13 had COPD, 14 had asthma, seven had ILD, two had ABPA, one had pleural thickening with enfolded lung, one had lung non-mucinous adenocarcinoma and one had primary ciliary dyskinesia. Three patients had no pre-existing lung condition and disease status was missing for five patients. 15 patients underwent antibiotic treatment during the study period.

**FIGURE 7:**
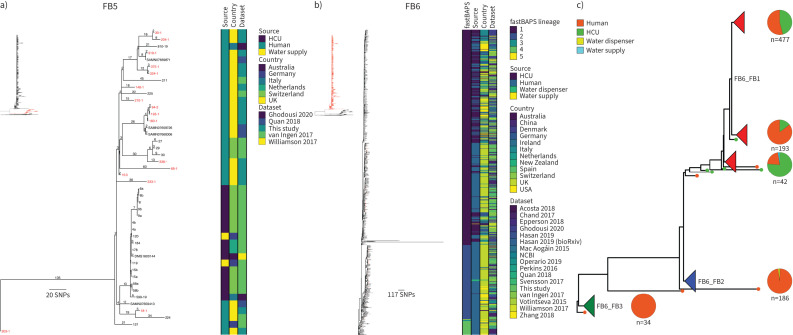
*Mycobacterium chimaera* global fastBAPS cluster phylogenies. Rooted maximum likelihood phylogenetic trees for a) *M. chimaera* FB5 (n=45), b) *M. chimaera* FB6 (n=765) and c) *M. chimaera* FB6 with major clades collapsed (pie charts are shown for each major clade showing the distribution of sources for that clade). The taxa are clustered according to their sequence similarities. The lengths of the branches are scaled in nucleotide substitutions per site. The source of isolate, country of collection and study are shown as datastrips to the right of the phylogenies. Isolates from the Royal Brompton Hospital are shown in red colour. Scale bars shown in single nucleotide polymorphisms (SNPs) per site. Details of the study datasets are provided in supplementary text S1 and supplementary file S3. HCU: heater-cooler unit; NCBI: National Center for Biotechnology Information.

**FIGURE 8:**
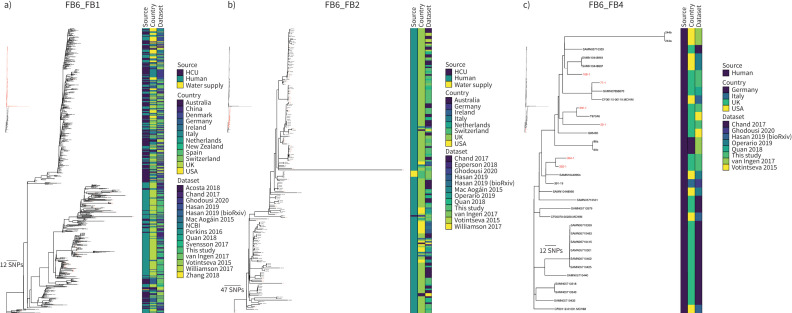
*Mycobacterium chimaera* fastBAPS cluster FB6 phylogenies. Maximum likelihood phylogenetic trees for a) FB6_FB1 (n=540), b) FB6_FB2 (n=186) and c) FB6_FB4 (n=34). The taxa are clustered according to their sequence similarities. The lengths of the branches are scaled in nucleotide substitutions per site. The source of isolate, country of collection and study are shown as datastrips to the right of the phylogenies. Isolates from the Royal Brompton Hospital are shown in red colour. Scale bars shown in single nucleotide polymorphisms (SNPs) per site. Details of the study datasets are provided in supplementary text S1 and supplementary file S3. HCU: heater-cooler unit; NCBI: National Center for Biotechnology Information.

The global phylogenetic tree containing 155 isolates from Royal Brompton Hospital and 671 previously published isolates was topologically similar to the tree built using only the isolates from the Royal Brompton Hospital with a single major clade with low genetic diversity present, Mc_FB6 (Group 1) (figure 6c). Isolates from the two clusters with more than 10 representatives, Mc_FB5 (Group 2) and Mc_FB6, were remapped to local references and new phylogenetic trees constructed (figure 7a and b). Most isolates in Mc_FB5 were collected from patients with respiratory conditions or patients with infections following cardiac surgeries. There was, however, a distinct subclade rooting within this diversity containing only isolates from HCUs or hospital water supplies (figure 7a). Within Mc_FB6 a more deeply rooting (ancestral) diverse clade contained mainly human isolates and the larger shallower clade rooting within this contained most of the HCU isolates interspersed with human isolates (figure 7b and c). Due to the large number of isolates assigned to Mc_FB6 (n=765), further genomic lineage assignment was performed using fastBAPS to identify five sublineages within Mc_FB6 (figure 7b and c). New mapping was performed for the three lineages with more than 10 isolates (figure 8a–c). Two of these lineages, Mc_FB6_FB2 and Mc_FB6_FB4, were completely or nearly completely composed of patient isolates (figure 8b and c). Most of the isolates collected from HCUs or water supplies made up the bulk of Mc_FB6_FB1, although human isolates were distributed throughout the tree (figure 8a). Putative transmission clusters were calculated for all five lineages using a pairwise SNP threshold of 30 SNPs (supplementary table S7). Transmission clusters containing Royal Brompton Hospital isolates were found in each of the lineages, with the largest found in Mc_FB6_FB1, comprising 258 HCU isolates, 230 patient isolates and a single isolate from a water supply (supplementary table S7).

## Discussion

The aims of this study were to characterise the population structure of MAC isolates collected in a London hospital, and to identify potential transmission between CF and non-CF patients. During the sampling period we identified 45 patients infected with more than one species or lineage. This suggests that many of the patients had polymicrobial infections and that certain lineages were preferentially sampled at different times. This has been observed previously in other longitudinal samples of patients with MAC lung disease [26] and suggests that single colonies from sputum swabs may be underrepresenting the underlying diversity of MAC species in patients. Therefore, to better understand the ecology and transmission of MAC species in the lung, a deep-sequencing approach in which plate sweeps from swabs or mycobacteria growth indicator tube cultures are utilised should be considered for future studies.

Previous studies have used thresholds of 15–25 SNPs to identify putative transmission clusters in NTM species [14, 19, 24]. Rather than using a previously applied or arbitrary threshold, we used a method developed to define SNP thresholds in *Staphylococcus aureus* [27]. This allowed us to calculate a threshold for each species using the longitudinal isolates collected in the Royal Brompton Hospital. The thresholds calculated for *M. intracellulare* and *M. avium* subsp. *hominissuis* of 16 SNPs were similar to those previously applied [24]. While we obtained higher thresholds of 30 and 58 SNPs for *M. chimaera* and *M. avium* subsp. *avium*, respectively, this did not result in an inflated number of transmission clusters, as applying a threshold of 16 SNPs to the *M. avium* subsp. *avium* dataset would have resulted in only two fewer clusters. These larger values were predominantly due to high levels of within-host diversity observed in patients with both *M. avium* subsp. *avium* and *M. chimaera*.

Using these SNP thresholds, we were able to identify putative transmission clusters among all four of the MAC species/subspecies investigated. Broadly, two different patterns of infection were observed. Among *M. intracellulare* and the two *M. avium* subspecies, our results suggested that there have been multiple independent introductions into the human population followed by onward transmission. Our analysis of *M. chimaera*, however, identified a single large transmission cluster which linked 106 out of 155 (68.4%) of our patients infected with this species. Most of these transmission clusters contained both CF and non-CF patients, and a small number also contained patients with no pre-existing lung conditions.

Using our criteria, we were unable to identify potential epidemiological links for most patients included in the transmission clusters. The exception to this was the largest *M. chimaera* transmission cluster where we found 12 potential epidemiological links up to 1 year before sampling began. The tight infection control surrounding patients with CF, which prioritises preventing cross-infection through hygiene and segregation, means that they are unlikely to have interacted with patients with other lung conditions while in hospital. Outpatient clinics are organised so that patients are seen individually and, during inpatient care, CF patients are given individual rooms with *en suite* facilities. This implies that transmission is likely occurring *via* other pathways, such as through environmental intermediates or through a reservoir of healthy, asymptomatic carriers in the wider population. This observation is not unique and there has been considerable debate whether NTM transmission between patients is occurring, even when transmission is strongly supported by genome-based analysis [14, 19, 28]. The limited evidence of epidemiological links between patients in transmission clusters as well as the ubiquitous presence of NTM species in the environment, especially water supplies, has led some researchers to suggest that the dissemination of NTM lineages at a national level is associated with exposure to contaminated water supplies [19]. While there is certainly strong evidence for the same lineages being isolated from water supplies and patients in the same location [24], the absence of a single national water supply in even a comparatively small country like the UK would suggest that closely related isolates collected from different geographical locations are unlikely to be due to a single contaminated water supply. It is, however, possible that transmission networks may include local water supplies as intermediates. The alternative hypothesis that NTM populations are being maintained in apparently healthy individuals with no symptoms of NTM lung disease is equally worthy of consideration. This would explain the presence of local and long-distance transmission clusters in the absence of direct epidemiological links as well as the presence of phylogeographical structure in NTM trees. To better address how NTM species are being transmitted, future studies should focus on collecting isolates from the environment as well as from the general population, perhaps focusing on smokers where there is some evidence that they may provide a reservoir for NTM species and other opportunistic pathogens [20].

The inclusion of published genomes alongside the Royal Brompton Hospital isolates allowed us to use our defined pairwise SNP thresholds to identify putative MAC lineages circulating in the UK and globally. Apart from the *M. chimaera* lineages that were known to be associated with HCUs, we were unable to identify large globally circulating lineages like those observed in *M. abscessus*. This potentially suggests different evolutionary dynamics among the MAC species or, in the case of *M. intracellulare*, may simply reflect a lack of comprehensive sampling. We were only able to identify a single lineage containing Royal Brompton Hospital isolates among the two *M. avium* subspecies that included isolates from outside the UK. This cluster comprised most of the isolates in MAA_FB15 (figure 4b). Given that most *M. avium* genomes have been collected in the UK or USA, a more comprehensive global sampling strategy could potentially reveal the existence of additional globally circulating clones.

When we calculated global transmission clusters for *M. chimaera* using a pairwise SNP threshold of 30 SNPs (supplementary table S7), we found that most clusters either consisted of isolates from across Europe or included isolates from other parts of the world such as Australia. Notably, we identified a single large transmission cluster containing 489 isolates from 12 countries that included most of the HCU-associated isolates. Previous work investigating the global population structure of *M. chimaera* suggested that the majority of *M. chimaera* transmission globally was associated with point-source contamination of HCUs during manufacturing which then led to direct infection of patients undergoing cardiac surgery [11]. Our study included isolates collected from HCUs and cardiac patients as well as patients with lung conditions such as CF and non-CF bronchiectasis. This enabled us to investigate the global population structure of *M. chimaera* in more detail than has previously been possible [11, 29]. Strikingly, we discovered clear evidence for distinct lineages within the global phylogeny that were almost exclusively associated with patient isolates, indicating considerable onward transmission. In addition, most of the HCU-associated isolates were clustered together in a single lineage that was derived from the lineages containing only human isolates (figure 6b). This was also true of a second, smaller, lineage that contained only HCU and environmental isolates. The results suggest that the predominant HCU-associated lineage is descended from a *M. chimaera* population already circulating among patients with respiratory diseases, and that a similar derivation has happened on at least one other occasion.

Potential limitations of this study are the lack of environmental samples as well as the distinct bias with respect to country of isolation in the contextual dataset. Given the lack of direct patient–patient contact and epidemiological links, inclusion of samples collected from wards and hospital water supplies could potentially have revealed additional vectors of transmission. Assembling useful contextual datasets is reliant on what is available in the public domain, and the geographical bias towards the USA and UK in these datasets is not confined to this study.

Our results showed that polymicrobial infections by different MAC species are not uncommon and that transmission is occurring between CF and non-CF patients even in the presence of strict infection controls. This suggests that all of the species and subspecies within the MAC, like *M. abscessus*, are capable of generating lineages that can sustain transmission within the human population, albeit to different extents. We also provided evidence that the lineage responsible for the HCU-vectored *M. chimaera* outbreak was derived from an existing lineage which was already circulating among patients with pre-existing lung conditions, indicating that this is also true of *M. chimaera*, even after excluding the extensive HCU-vectored outbreak event. To better understand the transmission dynamics and to prevent continuing circulation of MAC and other NTM species, future studies should include sampling of environmental reservoirs and potential asymptomatic carriers.

## Supplementary material

10.1183/13993003.01237-2022.Supp1 **Please note:** supplementary material is not edited by the Editorial Office, and is uploaded as it has been supplied by the author.
Supplementary text S1, supplementary tables 1-7, supplementary figures 1-4 erj-01237-2022.supplement1
Supplementary file 2 erj-01237-2022.supplement2
Supplementary file 3 erj-01237-2022.supplement3

## Shareable PDF

10.1183/13993003.01237-2022.Shareable1 This one-page PDF can be shared freely online.
Shareable PDF ERJ-01237-2022.Shareable
