# Supplementary material for: Mycobacterium avium complex genomics and transmission in a London hospital
Source: Eur Respir J. 2023 Apr 20;61(4):2201237. doi: 10.1183/13993003.01237-2022 (PMC10116071; doi:10.1183/13993003.01237-2022)
Supplement: Supplementary file 1 [file erj-01237-2022.supplement1.pdf]

Supplementary Text S1 - Detailed methods

Consent/ethics

The study (access to patients' clinical data) was approved by the NHS Health Research Authority (HRA) and Health and Care Research Wales (HCRW) (REC reference 21/HRA/2554).

Culturing, DNA extraction and sequencing

Sputum samples were grown in BBL MGIT media (Becton Dickinson; BD) in a BACTEC MGIT 960 (BD) culture system until the system indicated likely Mycobacterial growth. NTM species confirmation was performed using the GenoTypeMycobacterium CM VER 2.0 (Hain Lifescience) System. Confirmed MAC cultures were regrown from bead stock cultures in BBL MGIT media (BD) in a BACTEC MGIT 960 (BD) culture system until the system indicated growth. In the absence of growth, DNA was extracted from the bead stock. DNA extractions were performed as previously described (https://dx.doi.org/10.17504/protocols.io.bf28jqhw). A total of 1189 DNA extracts were sequenced by the core pipeline teams at the Wellcome Sanger Institute.

Clinical data pertaining to patients from whom NTM cultures were isolated were collected from electronic health records at the RBH. Data included patients' sex, age at the time of first positive NTM culture, height, weight, lung function test results, comorbidities, medication history and date of death (where applicable). Anonymization was undertaken by removing personal data, including patients' hospital numbers, prior to analysis.

Sequence QC, mapping and phylogenetics

Basic quality control metrics for the raw sequence data were generated using FastQC v0.11.9 [1]. Sequence reads with similarity to *Mycobacterium* species were identified using Kraken v0.10.6 [2] and Bracken v1.0 [3]. Samples with < 70% reads mapping to a *Mycobacterium* species were excluded from further analyses (n = 116). Seven isolates not belonging to the MAC (*M. abscessus, M. chelonae, M. simiae)* were removed from the dataset. Sequence reads for each species were trimmed using Trimmomatic v0.33 [4] and mapped to appropriate references (supplementary table 1) using BWA mem v0.7.17 (minimum and maximum insert sizes of 50 bp and 1000 bp respectively) [5]. Single nucleotide polymorphisms (SNPs) were called using SAMtools v1.2 mpileup and BCFtools v1.2 (minimum base call quality of 50 and minimum root squared mapping quality of 30) as previously described [6]. Samples with reads that mapped to < 80% of the reference were excluded (n = 70). Variant sites were extracted from the resulting alignments using snp-sites v2.5.1 [7]. Whole species maximum likelihood phylogenetic trees were built using IQ-tree v1.6.5 accounting for constant sites (-fconst; determined using snp-sites -C) with the built-in model testing (-m MFP) to determine the best phylogenetic model and 1000 ultrafast bootstraps (-bb 1000) [8].

For higher-resolution phylogenies within fastBAPS lineages, recombinant regions were identified and removed from alignments using GUBBINS [9] and new phylogenetic trees were constructed as described above. Pairwise SNP distances were calculated for all pairs of isolates using pairsnp [10].

Global collections

To provide context for each the isolates sequenced for each species in this study, datasets consisting of published sequenced isolates were assembled (Supplementary File 3) [11–31]. Sequence data were downloaded from the European Nucleotide Archive (ENA) and trimmed;

Sample QC, mapping and phylogenetic tree construction were performed as detailed above. Only the first isolate from each patient was included from the RBH isolates for each species/subspecies.

Genome assemblies

A previously published pipeline was used to produce annotated assemblies [32]. Briefly, sequence reads were assembled with spades v 3.10.10 [33] and assemblies were improved by first scaffolding the assembled contigs using SSPACE v2.0 [34] and filling the sequence gaps with GapFiller v1.11 [35].

Transmission and epidemiological linkage

Genomic lineages were identified using fastBAPS [36] and new alignments were created for lineages ≥ ten isolates by aligning sequence reads for included isolates against the assembly that had the smallest number of contigs (using the method described above). In order to calculate a pairwise SNP threshold to determine putative transmission clusters within each genomic lineage, pairwise SNP distances for all isolates for each species in the RBH datasets were calculated. Using a previously described method [37], the transmission threshold for each species, regardless of lineage, was calculated by taking the 95th percentile of the maximum within-patient isolate pairwise SNP distances for all patients and adding twice the number of mutations expected to occur in a six month period. To account for excess within-patient diversity observed in the *M. chimaera* FB1 and *M. avium* subsp. *avium* FB14 lineages (Fig A in S1 Data), pairwise SNP distances greater than 25 and 50 (assumed to result from infection with multiple lineages) were removed respectively before the above calculations were performed. Based on these results, the R library iGRAPH [38,39] and pairwise SNP thresholds of 16 (*M. intracellulare* and *M. avium* subsp. *hominissuis*), 30 (*M. chimaera*) and

58 SNPs (*M. avium* subsp. *avium*) were used to calculate putative transmission clusters in each genomic lineage. Finally, in order to identify possible epidemiological links between patients infected within the same transmission clusters, hospital stay records were examined for epidemiological contacts. The latter were defined as patients attending the same ward on the same day up to one year prior to the collection of the first sequenced isolate.

References

1. Babraham Bioinformatics. FastQC A Quality Control tool for High Throughput Sequence Data. www.bioinformatics.babraham.ac.uk/projects/fastqc/ Date last updated: December 14 2021. Date last accessed: December 14 2021.

2. Wood DE, Salzberg SL. Kraken: ultrafast metagenomic sequence classification using exact alignments. Genome Biol 2014; 15: R46.

3. Lu J, Breitwieser FP, Thielen P *et al*. Bracken: estimating species abundance in metagenomics data. PeerJ Comput Sci 2017; 3: e104.

4. Bolger AM, Lohse M, Usadel B. Trimmomatic: a flexible trimmer for Illumina sequence data. Bioinformatics 2014; 30: 2114–20.

5. Li H, Durbin R. Fast and accurate short read alignment with Burrows-Wheeler transform. Bioinformatics 2009; 25: 1754–60.

6. Harris SR, Feil EJ, Holden MTG *et al*. Evolution of MRSA during hospital transmission and intercontinental spread. Science 2010; 327: 469–74.

7. Page AJ, Taylor B, Delaney AJ *et al*. SNP-sites: rapid efficient extraction of SNPs from multi-FASTA alignments. Microb Genomics 2016; 2: e000056.

8. Nguyen L-T, Schmidt HA, von Haeseler A, *et al*. IQ-TREE: a fast and effective stochastic algorithm for estimating maximum-likelihood phylogenies. Mol Biol Evol 2015; 32: 268–74.

9. Croucher NJ, Page AJ, Connor TR *et al*. Rapid phylogenetic analysis of large samples of recombinant bacterial whole genome sequences using Gubbins. Nucleic Acids Res 2015; 43: e15.

10. Tonkin-Hill G. pairsnp. https://github.com/gtonkinhill/pairsnp. Date last updated: December 2021. Date last accessed: December 14 2021 .

11. van Ingen J, Kohl TA, Kranzer K *et al*. Global outbreak of severe *Mycobacterium chimaera* disease after cardiac surgery: a molecular epidemiological study. Lancet Infect Dis 2017; 17: 1033–41.

12. Caverly LJ, Spilker T, LiPuma JJ. Complete Genome Sequence of *Mycobacterium abscessus* subsp. *bolletii*. Genome Announc 2016; 4: e00543-16.

13. Hasan NA, Davidson RM, Epperson LE *et al.* Population Genomics of Nontuberculous Mycobacteria Recovered from United States Cystic Fibrosis Patients. bioRxiv 2019; 663559.

14. Bryant JM, Thibault VC, Smith DGE *et al*. Phylogenomic exploration of the relationships between strains of *Mycobacterium avium* subspecies *paratuberculosis*. BMC Genomics 2016; 17: 79.

15. Lande L, Alexander DC, Wallace RJ *et al*. *Mycobacterium avium* in Community and Household Water, Suburban Philadelphia, Pennsylvania, USA, 2010-2012. Emerg Infect Dis 2019; 25: 473–81.

16. Yoon J-K, Kim TS, Kim J-I *et al*. Whole genome sequencing of Nontuberculous Mycobacterium (NTM) isolates from sputum specimens of co-habiting patients with NTM pulmonary disease and NTM isolates from their environment. BMC Genomics 2020; 21: 322.

17. Votintseva AA, Pankhurst LJ, Anson LW *et al.* Mycobacterial DNA extraction for whole-genome sequencing from early positive liquid (MGIT) cultures. J Clin Microbiol 2015; 53: 1137–43.

18. Pfeiffer W, Braun J, Burchell J *et al*. Whole-genome analysis of mycobacteria from birds at the San Diego Zoo. PloS One 2017; 12: e0173464.

19. Quan TP, Bawa Z, Foster D *et al*. Evaluation of Whole-Genome Sequencing for Mycobacterial Species Identification and Drug Susceptibility Testing in a Clinical Setting: a Large-Scale Prospective Assessment of Performance against Line Probe Assays and Phenotyping. J Clin Microbiol 2018; 56: e01480-17.

20. Operario DJ, Pholwat S, Koeppel AF *et al*. *Mycobacterium avium* Complex Diversity within Lung Disease, as Revealed by Whole-Genome Sequencing. Am J Respir Crit Care Med 2019; 200: 393–6.

21. Rivas AE, Hollinger C, Oehler DA *et al*. DIAGNOSIS AND MANAGEMENT OF MYCOBACTERIOSIS IN A COLONY OF LITTLE PENGUINS (EUDYPTULA MINOR). J Zoo Wildl Med Off Publ Am Assoc Zoo Vet 2019; 50: 427–36.

22. Chand M, Lamagni T, Kranzer K *et al*. Insidious Risk of Severe *Mycobacterium chimaera* Infection in Cardiac Surgery Patients. Clin Infect Dis Off Publ Infect Dis Soc Am 2017; 64: 335–42.

23. Pankhurst LJ, Del Ojo Elias C, Votintseva AA *et al.* Rapid, comprehensive, and affordable mycobacterial diagnosis with whole-genome sequencing: a prospective study. Lancet Respir Med 2016; 4: 49–58.

24. Epperson LE, Timke M, Hasan NA *et al*. Evaluation of a Novel MALDI Biotyper Algorithm to Distinguish *Mycobacterium intracellulare* From *Mycobacterium chimaera*. Front Microbiol 2018; 9: 3140.

25. Acosta F, Pérez-Lago L, Ruiz Serrano MJ *et al*. Fast update of undetected *Mycobacterium chimaera* infections to reveal unsuspected cases. J Hosp Infect 2018; 100: 451–5.

26. Ghodousi A, Borroni E, Peracchi M *et al*. Genomic analysis of cardiac surgery-associated *Mycobacterium chimaera* infections in Italy. PloS One 2020; 15: e0239273.

27. Mac Aogáin M, Roycroft E, Raftery P *et al*. Draft Genome Sequences of Three *Mycobacterium chimaera* Respiratory Isolates. Genome Announc 2015; 3: e01409-15.

28. Perkins KM, Lawsin A, Hasan NA *et al*. Notes from the Field: *Mycobacterium chimaera* Contamination of Heater-Cooler Devices Used in Cardiac Surgery - United States. MMWR Morb Mortal Wkly Rep 2016; 65: 1117–8.

29. Svensson E, Jensen ET, Rasmussen EM *et al*. *Mycobacterium chimaera* in Heater-Cooler Units in Denmark Related to Isolates from the United States and United Kingdom. Emerg Infect Dis 2017; 23: 507–9.

30. Williamson D, Howden B, Stinear T. *Mycobacterium chimaera* Spread from Heating and Cooling Units in Heart Surgery. N Engl J Med 2017; 376: 600–2.

31. Zhang X, Lin J, Feng Y *et al*. Identification of *Mycobacterium chimaera* in heater-cooler units in China. Sci Rep 2018; 8: 7843.

32. Page AJ, De Silva N, Hunt M *et al.* Robust high-throughput prokaryote de novo assembly and improvement pipeline for Illumina data. Microb Genomics 2016; 2: e000083.

33. Bankevich A, Nurk S, Antipov D *et al*. SPAdes: a new genome assembly algorithm and its applications to single-cell sequencing. J Comput Biol J Comput Mol Cell Biol 2012; 19:455–77.

34. Boetzer M, Henkel CV, Jansen HJ *et al*. Scaffolding pre-assembled contigs using SSPACE. Bioinformatics 2011; 27: 578–9.

35. Boetzer M, Pirovano W. Toward almost closed genomes with GapFiller. Genome Biol 2012; 13: R56.

36. Tonkin-Hill G, Lees JA, Bentley SD *et al*. Fast hierarchical Bayesian analysis of population structure. Nucleic Acids Res 2019; 47: 5539–49.

37. Coll F, Raven KE, Knight GM *et al*. Definition of a genetic relatedness cutoff to exclude recent transmission of meticillin-resistant *Staphylococcus aureus*: a genomic epidemiology analysis. Lancet Microbe 2020; 1: e328–35.

38. R Core Team. R: A language and environment for statistical computing. 2013.

39. Csardi G, Nepusz T. The Igraph Software Package for Complex Network Research. InterJournal 2005; Complex Systems:1695.

Supplementary Table 1. Reference genomes used in this study.

Species/ subspecies	Reference	Length (Mb)	Country of isolation	Year(s) of isolation
M. intracellulare	ATCC 13950	5.4	South Korea	2012
M. avium subsp. *avium*	104	5.5	USA	1983
M. avium subsp. *hominissuis*	TH135	5.0	Japan	2004-2008
M. chimaera	DSM 44623	5.9	Italy	1999-2003

Supplementary Table 2. *Mycobacterium intracellulare* transmission clusters

fastBAPS cluster	Transmission cluster	Patients (n)	Bronchiectasis (n)	Cystic Fibrosis (n)	Other lung conditions (n)	No pre-existing lung condition (n)	Patients undergoing treatment (n)
Mi_FB2	Mi_FB2_1	2	0	2	0	0	1
Mi_FB3	Mi_FB3_1	16	8	7	1	0	4
Mi_FB5	Mi_FB5_1	3	2	0	1	0	2

Supplementary Table 3. *M. avium* subsp. *avium* transmission clusters

fastBAPS cluster	Transmission cluster	Patients (n)	Bronchiectasis (n)	Cystic Fibrosis (n)	Other lung conditio	No pre-existing lung	Patients undergoing

					ns (n)	condition (n)	treatment (n)
MAA_FB5	MAA_FB5_1	10	6	2	2	0	4
MAA_FB5	MAA_FB5_4	2	1	1	0	0	0
MAA_FB5	MAA_FB5_5	2	1	0	1	0	1
MAA_FB5	MAA_FB5_12	2	1	1	0	0	1
MAA_FB7	MAA_FB7_1	2	2	0	0	0	1
MAA_FB10	MAA_FB10_1	2	0	2	0	0	0
MAA_FB14	MAA_FB14_1	2	2	0	0	0	1

Supplementary Table 4. *M. avium* subsp. *hominissuis* transmission clusters

fastBAPS cluster	Transmission cluster	Patients (n)	Bronchiectasis (n)	Cystic Fibrosis (n)	Other lung conditions (n)	No pre-existing lung condition (n)	Patients undergoing treatment (n)
MAH_FB	MAH_FB8_	16	7	4	3	1	0

8	1						
MAH_FB10	MAH_FB10_1	4	2	2	0	0	1
MAH_FB11	MAH_FB11_1	6	3	1	2	0	0
MAH_FB12	MAH_FB12_2	4	1	2	1	0	2
MAH_FB14	MAH_FB14_3	4	2	2	0	0	0
MAH_FB14	MAH_FB14_7	2	0	2	0	0	0
MAH_FB14	MAH_FB14_9	7	3	2	1	0	1

Supplementary Table 5. *Mycobacterium chimaera* transmission clusters

fastBAPS cluster	Transmission cluster	Patients (n)	Bronchiectasis (n)	Cystic Fibrosis (n)	Other lung conditions (n)	No pre-existing lung condition (n)	Patients undergoing treatment (n)
Mc_FB3	Mc_FB3_1	106	43	24	25	3	15
Mc_FB3	Mc_FB3_3	2	1	1	0	0	0
Mc_FB3	Mc_FB3_4	2	2	0	0	0	0
Mc_FB3	Mc_FB3_6	2	1	0	1	0	0
Mc_FB3	Mc_FB3_1	4	1	1	1	1	1

	3						
Mc_FB3	Mc_FB3_16	2	0	0	1	1	1
Mc_FB3	Mc_FB3_17	2	1	1	0	0	0
Mc_FB3	Mc_FB3_19	2	0	1	0	1	0
Mc_FB3	Mc_FB3_25	2	1	1	0	0	1
Mc_FB3	Mc_FB3_30	4	0	1	0	0	0
Mc_FB4	Mc_FB4_1	2	1	1	0	0	1
Mc_FB4	Mc_FB4_2	9	3	3	3	0	2
Mc_FB4	Mc_FB4_3	6	1	2	2	0	1

Supplementary Table 6. *Mycobacterium chimaera* epidemiological links (n =15 patients)

Patients with epidemiological link	Hospital ward	Dates of overlapping stay
24/218	FOUL	02/02/12-12/02/12
163/218	FOUL	13/02/12
175/218	FOUL	05/04/12-16/04/12
130/218	LIND	30/04/12
76/218	FOUL	06/06/12-11/06/12
32/163	LIND	29/06/12

79/241	LIND	05/07/12
241/272	LIND	06/07/12
177/186	LIND	06/08/12
122/175/306	FOUL	25/10/12-30/10/12
30/122	FOUL	05/11/12-06/11/12
30/218	FOUL	12/11/12-13/11/12

Supplementary Table 7. *Mycobacterium chimaera* global transmission clusters containing RBH isolates

fastBAPS cluster	Transmission cluster	Total isolates (n)	Patients (n)	HCU (n)	Other (n)
FB5	FB5_1	21	4	15	2
FB5	FB5_2	7	7	0	0
FB5	FB5_3	5	5	0	0
FB5	FB5_6	4	4	0	0
FB6_FB1	FB6_FB1_1	489	230	258	1
FB6_FB1	FB6_FB1_2	6	6	0	0
FB6_FB1	FB6_FB1_8	5	5	0	0
FB6_FB1	FB6_FB1_21	4	4	0	0
FB6_FB2	FB6_FB2_1	22	22	0	0
FB6_FB2	FB6_FB2_2	36	36	0	0
FB6_FB2	FB6_FB2_8	16	16	0	0
FB6_FB2	FB6_FB2_17	10	10	0	0

FB6_FB2	FB6_FB2_32	11	11	0	0
FB6_FB2	FB6_FB2_35	2	2	0	0
FB6_FB4	FB6_FB4_8	4	4	0	0
FB6_FB4	FB6_FB4_11	3	3	0	0
FB6_FB4	FB6_FB4_16	2	2	0	0

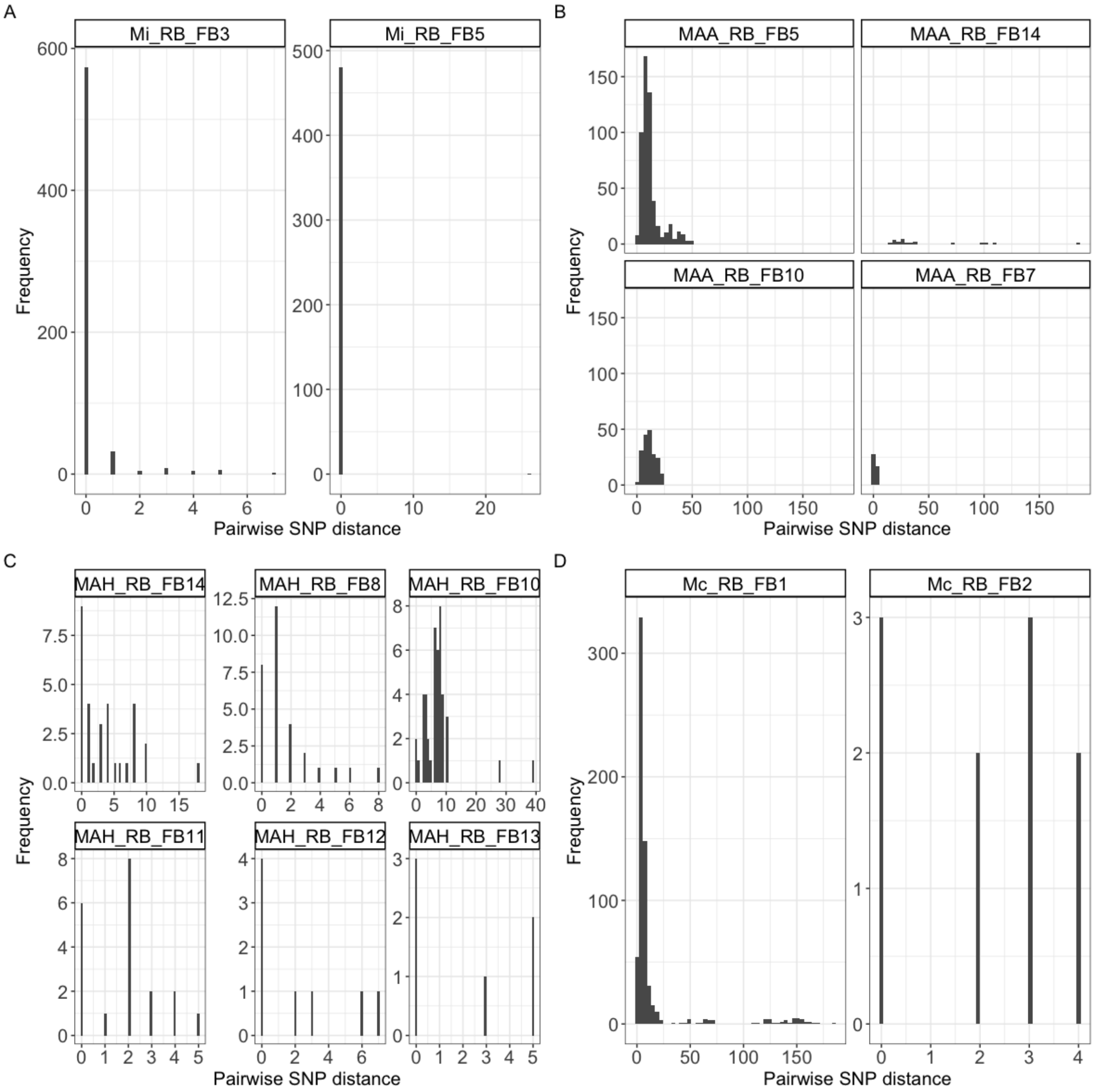

Supplementary Figure 1. Within-host isolate pairwise SNP diversity in fastBAPS lineages for A) *Mycobacterium intracellulare*; B) *Mycobacterium avium* subsp. *avium;* C) *Mycobacterium avium* subsp. *hominissuis* and D) *Mycobacterium chimaera*.

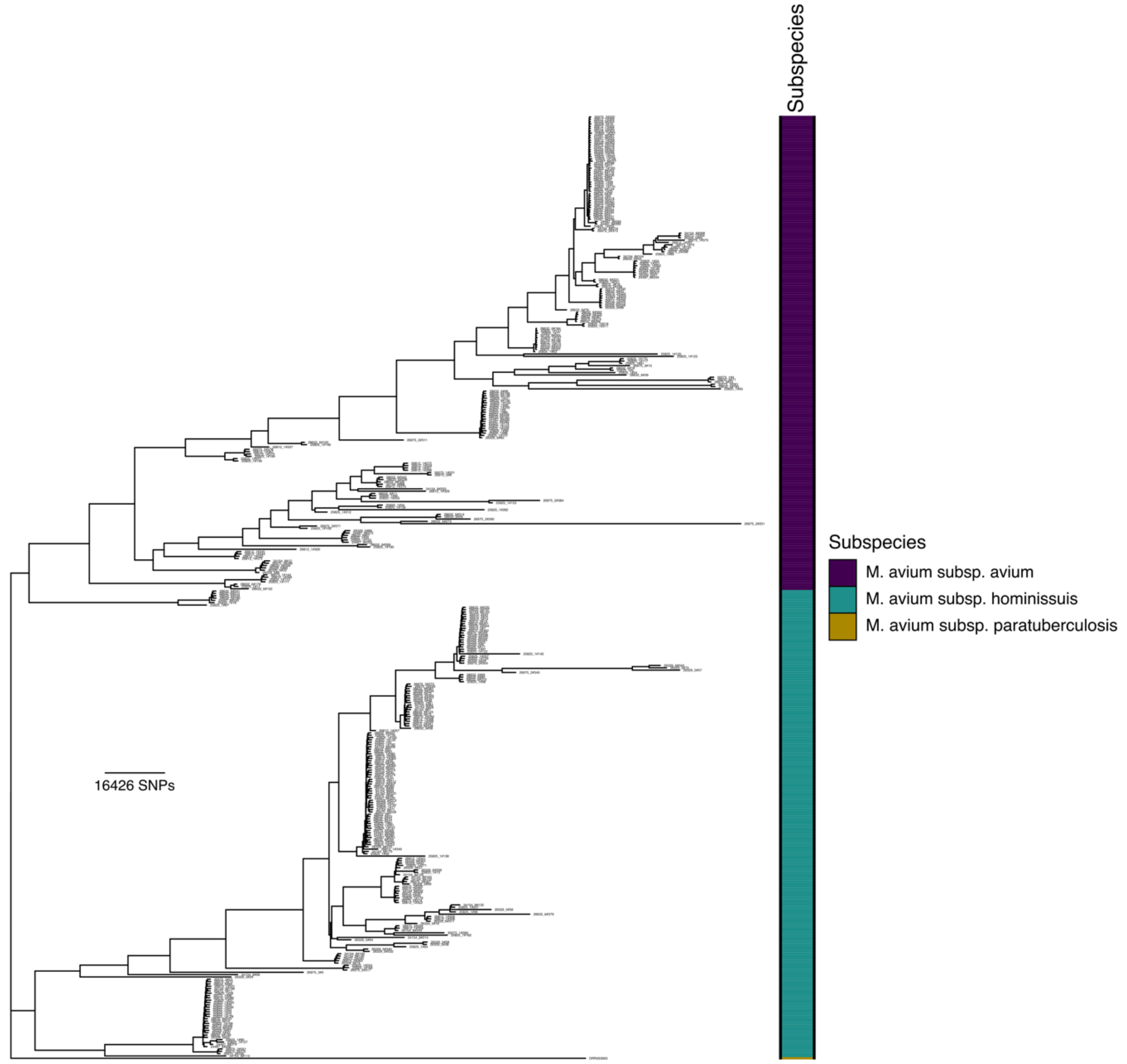

Supplementary Figure 2. Population structure of *Mycobacterium avium* at the Royal Brompton Hospital. Maximum likelihood phylogenetic tree of 406 *Mycobacterium avium* isolates rooted with a *Mycobacterium avium* subsp. *paratuberculosis* isolate (DRR263663).

The subspecies of each isolate is shown in the datastrip to the right of the phylogeny. The scale bar is shown in SNPs per site.

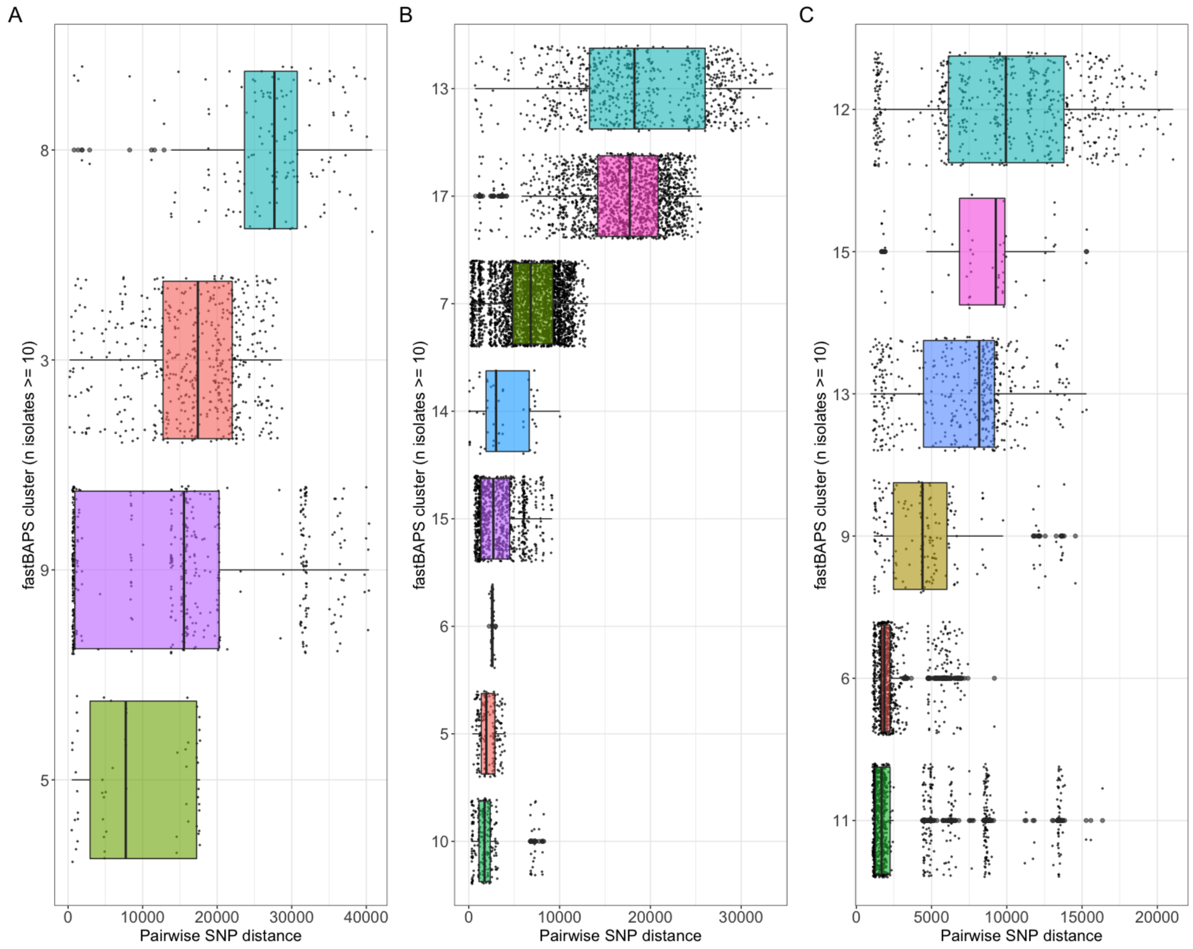

Supplementary Figure 3. Global fastBAPS cluster pairwise SNP distance distributions. Boxplots showing isolate pairwise SNP distances for A) *Mycobacterium intracellulare*; B) *Mycobacterium avium* subsp. *avium* and C) *Mycobacterium avium* subsp. *hominissuis*

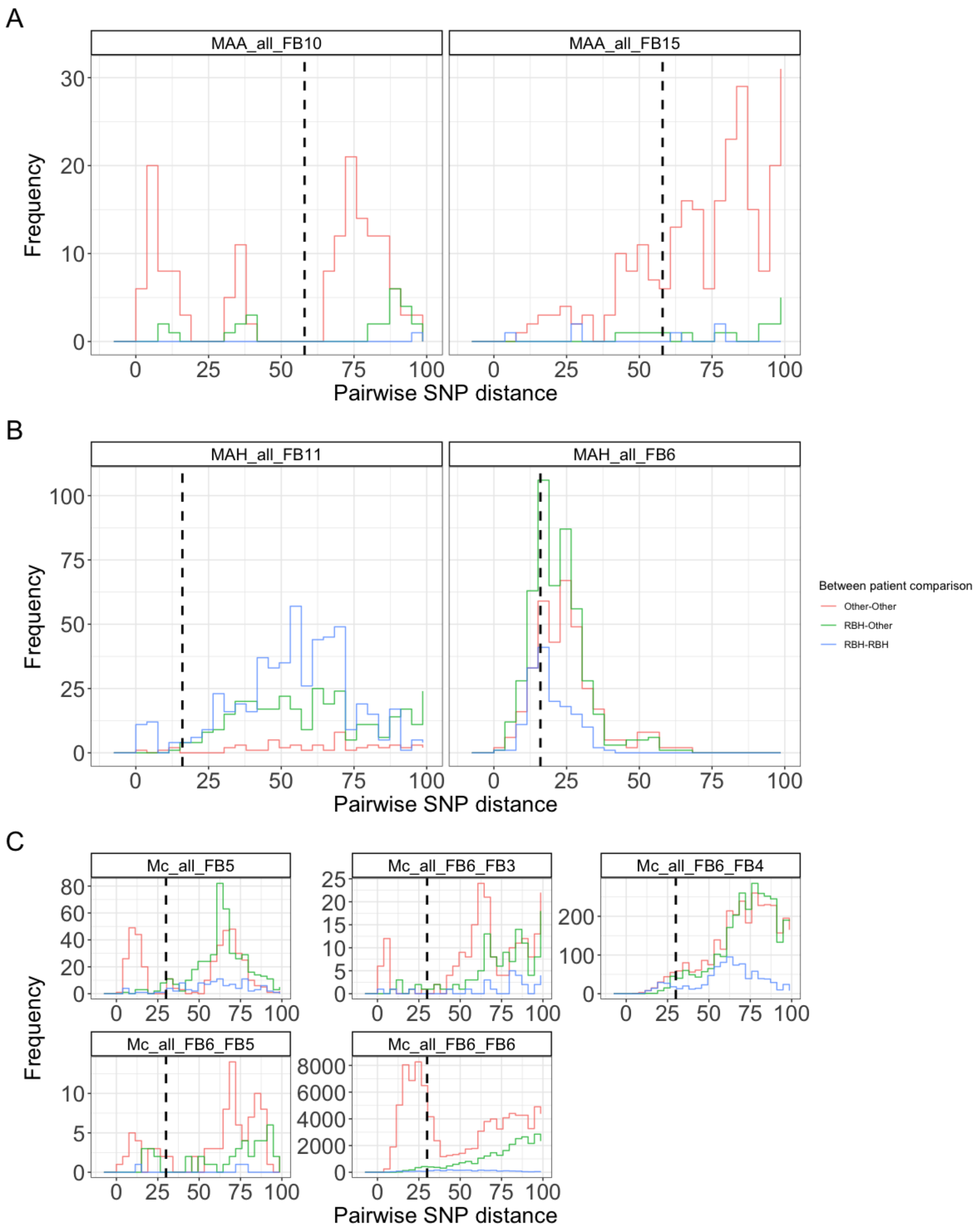

Supplementary Figure 4: Distribution of pairwise SNP distances between isolates from the Royal Brompton Hospital and other studies. Histograms showing distribution of

pairwise SNP distances for fastBAPS clusters for A) *Mycobacterium avium* subsp. *avium*; B) *Mycobacterium avium* subsp. *hominissuis* and C) *Mycobacterium chimaera*.
