# Supplementary material for: Mycobacterium avium complex genomics and transmission in a London hospital
Source: Eur Respir J. 2023 Apr 20;61(4):2201237. doi: 10.1183/13993003.01237-2022 (PMC10116071; doi:10.1183/13993003.01237-2022)
Supplement: Supplementary file 4 [file ERJ-01237-2022.Shareable.pdf]

EUROPEAN RESPIRATORY JOURNAL
ORIGINAL RESEARCH ARTICLE
A.J. VAN TONDER ET AL.

SHAREABLE PDF

Mycobacterium avium complex genomics and transmission in a London hospital

Andries J. van Tonder[1], Huw C. Ellis[2,3], Colin P. Churchward[3], Kartik Kumar[2,3], Newara Ramadan[4], Susan Benson[4], Julian Parkhill[1], Miriam F. Moffatt[3,5], Michael R. Loebinger[2,3,5] and William O.C. Cookson[2,3,5]

[1]Department of Veterinary Medicine, University of Cambridge, Cambridge, UK. [2]Host Defence Unit, Department of Respiratory Medicine, Royal Brompton Hospital, Guy's and St Thomas' NHS Foundation Trust, London, UK. [3]National Heart and Lung Institute, Imperial College London, London, UK. [4]Department of Microbiology, Royal Brompton Hospital, Guy's and St Thomas' NHS Foundation Trust, London, UK. [5]These three authors contributed equally.

Corresponding author: Julian Parkhill (jp369@cam.ac.uk)

Check for updates

Shareable abstract (@ERSpublications)
Transmission of *Mycobacterium avium* complex (MAC) species is indirect and may involve environmental intermediates or asymptomatic carriage in the wider population https://bit.ly/3FQWqnF

Cite this article as: van Tonder AJ, Ellis HC, Churchward CP, *et al*. *Mycobacterium avium* complex genomics and transmission in a London hospital. *Eur Respir J* 2023; 61: 2201237 [DOI: 10.1183/13993003.01237-2022].

This single-page version can be shared freely online.

Copyright ©The authors 2023.

This version is distributed under the terms of the Creative Commons Attribution Licence 4.0.

This article has an editorial commentary: https://doi.org/10.1183/13993003.00308-2023

Received: 22 June 2022
Accepted: 23 Oct 2022

Abstract

Background Non-tuberculous mycobacteria (NTM) are environmental microorganisms and opportunistic pathogens in individuals with pre-existing lung conditions such as cystic fibrosis (CF) and non-CF bronchiectasis. While recent studies of *Mycobacterium abscessus* have identified transmission within single CF centres as well as nationally and globally, transmission of other NTM species is less well studied.

Methods To investigate the potential for transmission of the *Mycobacterium avium* complex (MAC) we sequenced 996 isolates from 354 CF and non-CF patients at the Royal Brompton Hospital (London, UK; collected 2013–2016) and analysed them in a global context. Epidemiological links were identified from patient records. Previously published genomes were used to characterise global population structures.

Results We identified putative transmission clusters in three MAC species, although few epidemiological links could be identified. For *M. avium*, lineages were largely limited to single countries, while for *Mycobacterium chimaera*, global transmission clusters previously associated with heater-cooler units (HCUs) were found. However, the immediate ancestor of the lineage causing the major HCU-associated outbreak was a lineage already circulating in patients.

Conclusions CF and non-CF patients shared transmission chains, although the lack of epidemiological links suggested that most transmission is indirect and may involve environmental intermediates or asymptomatic carriage in the wider population.
